# Microbial diversity and activity in the *Nematostella vectensis* holobiont: insights from 16S rRNA gene sequencing, isolate genomes, and a pilot-scale survey of gene expression

**DOI:** 10.3389/fmicb.2015.00818

**Published:** 2015-09-02

**Authors:** Jia Y. Har, Tim Helbig, Ju H. Lim, Samodha C. Fernando, Adam M. Reitzel, Kevin Penn, Janelle R. Thompson

**Affiliations:** ^1^Department of Civil and Environmental Engineering, Massachusetts Institute of TechnologyCambridge, MA, USA; ^2^Department of Biological Sciences, University of North Carolina at CharlotteCharlotte, NC, USA

**Keywords:** *Nematostella vectensis*, holobiont, cnidaria, microbiota, mixotrophy, phasins

## Abstract

We have characterized the molecular and genomic diversity of the microbiota of the starlet sea anemone *Nematostella vectensis*, a cnidarian model for comparative developmental and functional biology and a year-round inhabitant of temperate salt marshes. Molecular phylogenetic analysis of 16S rRNA gene clone libraries revealed four ribotypes associated with *N. vectensis* at multiple locations and times. These associates include two novel ribotypes within the ε-Proteobacterial order Campylobacterales and the Spirochetes, respectively, each sharing <85% identity with cultivated strains, and two γ-Proteobacterial ribotypes sharing >99% 16S rRNA identity with *Endozoicomonas elysicola* and *Pseudomonas oleovorans*, respectively. Species-specific PCR revealed that these populations persisted in *N. vectensis* asexually propagated under laboratory conditions. cDNA indicated expression of the Campylobacterales and *Endozoicomonas* 16S rRNA in anemones from Sippewissett Marsh, MA. A collection of bacteria from laboratory raised *N. vectensis* was dominated by isolates from *P. oleovorans* and *Rhizobium radiobacter*. Isolates from field-collected anemones revealed an association with *Limnobacter* and *Stappia* isolates. Genomic DNA sequencing was carried out on 10 cultured bacterial isolates representing field- and laboratory-associates, i.e., *Limnobacter* spp., *Stappia* spp., *P. oleovorans* and *R. radiobacter*. Genomes contained multiple genes identified as virulence (host-association) factors while *S. stellulata* and *L. thiooxidans* genomes revealed pathways for mixotrophic sulfur oxidation. A pilot metatranscriptome of laboratory-raised *N. vectensis* was compared to the isolate genomes and indicated expression of ORFs from *L. thiooxidans* with predicted functions of motility, nutrient scavenging (Fe and P), polyhydroxyalkanoate synthesis for carbon storage, and selective permeability (porins). We hypothesize that such activities may mediate acclimation and persistence of bacteria in a *N. vectensis* holobiont defined by both internal and external gradients of chemicals and nutrients in a dynamic coastal habitat.

## Introduction

Communities of microbes and their animal hosts are collectively known as holobionts (Rohwer et al., 2002). The microbial portion of the holobiont (i.e., the microbiota) contributes to the molecular and physiological functions of a wide diversity of hosts. For example, bacteria are known to breakdown complex plant-polymers and polysaccharides in termites (Xu et al., 2003; Warnecke et al., 2007; Mahowald et al., 2009), synthesize essential amino acids and vitamins in sharpshooter insects (Wu et al., 2006), aid the development of particular organs and systems in humans (Dobber et al., 1992; Rawls et al., 2004; O'Hara and Shanahan, 2006; Rader and Nyholm, 2012), and deter predators and pathogens in corals (Reshef et al., 2006; Bosch, 2013). Evidence that the composition and succession of the microbiota are species specific for particular animal hosts comes from the identification of mechanisms for interaction which appear to have diverged with host speciation (Rawls et al., 2006; Ley et al., 2008; Ryu et al., 2008; Fraune et al., 2010; Ochman et al., 2010).

Cnidarians are a focal taxonomic group in marine habitats for understanding the interaction between animals and microbes. Much work on cnidarian-microbe associations has focused on identifying bacterial species that might cause or prevent disease, particularly the various “band” diseases that are increasingly common in reef building corals (Bourne et al., 2009; Kimes et al., 2010; Mouchka et al., 2010). However, detailed functional connections between corals and bacteria remain unknown. Mechanistic studies using the hydrozoan *Hydra* have revealed species-specific bacterial communities and precise temporal regulation of the microbiome during its development (Fraune and Bosch, 2007; Franzenburg et al., 2013a,b). Together, these data from cnidarian species suggest that bacterial communities are integral and specific components to each cnidarian holobiont with a spectrum of functions.

**Figure 1:**
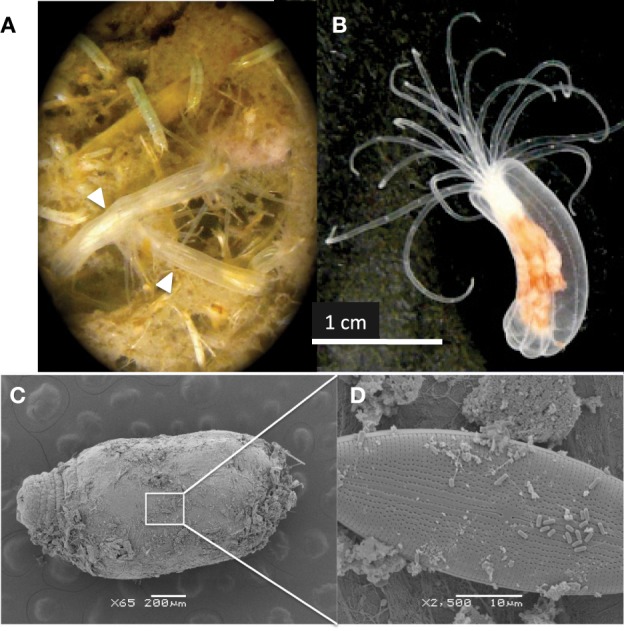
**(A)** Two *N. vectensis* anemones residing at the surface of a core of Sippewissett Marsh, MA (white arrows). Other invertebrates evident in image were also observed in the *N. vectensis* gut. **(B)** *N. vectensis* polyp maintained in the laboratory. **(C)** Scanning electron micrograph of *N. vectensis* collected from Sippewissett Marsh (October 2009) **(D)** higher magnification showing the presence of **(D)** diatoms and rod-shaped microorganisms on the anemone exterior.

Recently, the anthozoan *Nematostella vectensis* has been developed into a model organism for metazoan evolution and development due to its tractability in the lab, easily induced sexual and asexual reproduction and sequenced genome including a repertoire of predicted innate immunity genes (Putnam et al., 2007; Genikhovich and Technau, 2009; Renfer et al., 2010; Reitzel et al., 2012; Stefanik et al., 2013). A sedentary carnivore, this anemone resides exclusively in estuaries (Hand and Uhlinger, 1994) including those of extreme salinity (Sheader et al., 1997), temperature (Williams, 1983; Kneib, 1988; Reitzel et al., 2013) and sulfide fluxes (Howes et al., 1985). *N. vectensis* does not harbor zooxanthellae or any other known eukaryotic symbionts (Figure 1) and mainly preys on small free-living organisms in salt marshes, including copepods, midge larvae, worms (nematodes, polychaetes, and oligochaetes) and rotifers (Frank and Bleakney, 1978; personal observation) (Figure 1A).

As a first step to characterize the microbiota of *N. vectensis* both in the wild and under controlled laboratory conditions we have employed cultivation-independent analyses of 16S rRNA gene diversity including cloned sequence analysis, strain isolation, genome sequencing, and analysis of expressed RNAs to determine: (1) Whether *N. vectensis* is associated with similar populations of microorganisms in geographically distinct salt marshes and when transferred to laboratory cultures with artificial seawater conditions, (2) Whether these microbial populations are metabolically active within the host tissue, and (3) If associated microbes have specific genes that may promote survival in the holobiont environment. Taken together, the following data provide evidence that *N. vectensis* maintains interactions with populations of microorganisms, of which several appear to be active based on detection of expressed RNAs. Future work to determine the nature of these interactions will advance our understanding of how microorganisms contribute to the physiology and ecology of the anemone holobiont.

## Methods

### Anemone collection and maintenance

*N. vectensis* adults were collected from Sippewissett Marsh, Massachusetts USA (MA-I to MA-V, MA-II), Clinton, Connecticut USA (CT) and Mahone Bay, Nova Scotia Canada (MB) between July 2008 and March 2010, preserved in RNAlater (Ambion, Inc.) and stored at 4°C for DNA analysis. Before nucleic acid extraction, field anemones were directly removed from RNAlater and rinsed three times in deionized water. Sediment samples from the site of *N. vectensis* collection were retrieved from Sippewissett Marsh in November 2008 and June 2009 (Table 1). Four hundred and eighty milliliter marsh water was collected in June 2009 and filtered using Sterivex 0.22 micron cartridge filters (Millipore) on-site. The cartridges were kept on ice and then frozen in −20°C until DNA extraction.

**Table 1:** **Overview of sample collection and analysis**.

**Sample codes**	**Collection location (date)**	**Air temp. month^a^ (°C)**	**Water temp. (°C)**	**16S rRNA clone library**	**Species-specific PCR**	**cDNA**	**Isolates**
LAB I	MIT Laboratory (2008 and 2009)	21 to 23	21 to 23	√	√	√	√
MA-I	Sippewissett Marsh, MA (July, 2008)	16 to 31	22^b^	√			
CT	Clinton, CT (July, 2008)	15 to 34	ND	√			
MB	Mahone Bay, NS (Sept, 2008)	4 to 25	ND	√			
MA-II	Sippewissett Marsh, MA (Nov. 2008)	−7 to 20	6	√			
SED-MA-II	Sippewissett Marsh, MA (Nov. 2008)	−7 to 20	6	√			
MA-III	Sippewissett Marsh, MA (June, 2009)	5 to 28	20		√		
SED-MA-III	Sippewissett Marsh, MA (June, 2009)	5 to 28	20		√		
WATER-MA-III	Sippewissett Marsh, MA (June, 2009)	5 to 28	20		√		
MA-IV	Sippewissett Marsh, MA (July, 2009)	8 to 29	20	√		√	
				(cDNA)			
LAB II	MIT Laboratory (2010)	21 to 23	21 to 23				√
MA-V	Sippewissett Marsh, MA (March-April, 2010)	−8 to 20	ND				√

a *Monthly air temperature ranges were obtained from weather series of Cape Cod Air Station, MA (12 miles away from the Great Sippewissett Marsh), Clinton, CT, and Western Head, NS (42 miles from Mahone Bay, NS)*.

b *Water temperature was measured by an Onset® temperature logger. ND: Not Determined*.

Laboratory-acclimated *N. vectensis* were originally collected from Sippewissett Marsh, MA (summer of 2007, multiple trips) and were maintained at MIT for at least 6 months before DNA extraction. *N. vectensis* were kept in artificial seawater (ASW) adjusted to a salinity of 10 ppt (Instant Ocean, Spectrum Brands, Inc.) at room temperature (21–23°C) and fed *Artemia* nauplii three times a week over 6 months. To prepare laboratory-acclimated *N. vectensis* (LAB), individuals were transferred to autoclaved saline and fed with bleached *Artemia* nauplii for 4 weeks to reduce the effects of laboratory microbial contaminants on the host microbiota. Prior to genomic DNA extraction, laboratory anemones were incubated in autoclaved 10 ppt ASW for 2 days without feeding to eliminate digested food particles and rinsed three times in deionized water to remove loosely attached microbes and debris.

### Molecular diversity of the *N. vectensis* holobiont

#### Preparation and analysis of 16S rRNA gene clone libraries

Genomic DNA was extracted from whole anemones (*n* = 3 to 5 per extraction depending on anemone size) using the DNeasy® Blood and Tissue kit (Qiagen Sciences). DNA from sediment and water filters was extracted using UltraClean™ Soil DNA Isolation Kit (Mo Bio Laboratories, Inc.) according to manufacturer instructions. Blunt end 16S rRNA products were amplified with the universal bacterial PCR primers 27F (5′-AGA GTT TGA TCM TGG CTC AG-3′) and 805R (5′-GGA CTA CCA GGG TAT CTA ATC CC-3′) using Phusion polymerase (Finnzymes) under the following conditions: 1 cycle of 98°C for 30 s, 35 cycles of 98°C for 10 s, 52°C for 30 s, 72°C for 30 s and 1 cycle of 72°C for 10 min. Quadruplicate PCRs were carried out and pooled for each sample to reduce potential influence of PCR-generated mutations. PCR products were gel-purified (Qiagen kit), cloned into the pCR-Blunt vector (Invitrogen) and transformed into chemically competent TOP10 cells (Invitrogen) according to the manufacturer instructions. Ninety-six to 120 clones were randomly selected from each library and insert sequences were amplified using flanking primers M13F (5′TGT AAA ACG ACG GCC AGT) and M13R (5′-AGG AAA CAG CTA TGA CCA T-3′). PCR products were sequenced from the 27F primer using BigDye® Terminator v3.1 Cycle Sequencing Kit according to manufacturer recommendations on a 3130 Genetic Analyzer (ABI). Sequences were trimmed and sorted into operational taxanomic units (OTUs) defined by 99% nucleic acid identity using Sequencher 4.5 (Gene Codes Corporation). Sequences were checked for chimeras using Bellerophon (Huber et al., 2004) and Mallard (Ashelford et al., 2006). Non-chimeric clones were identified by querying the Genbank database using NCBI BLAST (Altschul et al., 1997). Representative clones for each 99% OTU were aligned to the SINA database in Silva (Pruesse et al., 2012; Quast et al., 2013) and their phylogenies were inferred using the neighbor-joining algorithm implemented in CLC Genomic Workbench (version 7).

#### Species-specific PCR

Species-specific PCR primers were designed for the ribotypes found in association with *N. vectensis* at different locations. Primers were designed using Primer-BLAST (Rozen and Skaletsky, 2000) and the annealing temperatures of the primers were optimized by incubation with negative controls of non-target organisms until no non-specific targets could be amplified. Primer sequences and optimized annealing temperatures (Ta) are as follows: Campylobacterales OTU, NVeps81F TAGCTTGCTAGAGTGTCAGC and NVeps677R TTTGTCTTGCAGTTCTATGGTTAA, Ta: 55°C; Spirochete-like OTU, Spiro165F GGGGTAATACCGAATGATCTA GG and Spiro655R TTCCAACGCAACAATACAGTTAAG, Ta: 57°C; *Endozoicomonas elysicola*, Endo80F AGCTTGCTCTTTGCCGACGAG and Endo624R CTTTCACATCCAACTTAGGT AGCC Ta: 58°C; *Pseudomonas oleovorans*, - Po23S-323F: GTACACGAAACGCTCTTATCAATG and Po23S-1475R AAATCAGCCTACCACCTTAAACAC, Ta: 57°C. The expected PCR product sizes were approximately 600, 490, 540, and 1150 bp, respectively. Ten nanogram of DNA from MA-III, SED-MA-III, WATER-MA-III, and LAB samples were used as templates for the species-specific PCR using Phusion (Finnzymes) using the same thermocycler profile as for the 16s universal primers (see above). Positive amplicons with correct size products (if any) were confirmed by cloning and sequencing or by restriction fragment length polymorphism (RFLP) analysis with the enzyme HaeIII.

### Isolation and characterization of bacterial strains from the *N. vectensis* holobiont

Microbial strains were isolated from *N. vectensis* collected from Sippewissett Marsh (multiple trips; March–May 2010) or from the laboratory-maintained stock (February 2008 and 2010). Bacterial isolation methods were varied in an effort to increase the diversity of microbial isolates. Anemones were washed with 1X PBS (with or without 50 ugmL^−1^ gentamycin treatment to inactivate surface-associated microbes depending on sample) for 1 h at room temperature, after which they were homogenized in 1X PBS using a flame-sterilized tissue grinder (Wheaton). Some anemones were treated with 5 mM Type 1 collagenase (Calbiochem) for 30 min at 37°C for further tissue maceration, prior to tissue grinding. Partial sequences of the 16S rRNA genes were obtained after screening by RFLP analysis with the HaeIII enzyme and compared to sequences recovered by clone library analysis. Isolates with ribotypes recovered from multiple individual anemones, geographic locations, and/or sampling times, were considered to have evidence of stable association with the anemone suggesting symbiosis or “the living together of unlike organisms” (de Bary, 1879). Stable associates were selected for physiological characterization and genome sequencing.

Physiological tests were conducted in triplicate to characterize particular attributes of each bacterial isolate isolated from *N. vectensis*. Heterotrophic growth media for physiological characterization consisted of 2216 marine broth or agar (Difco) with additional tests for general growth (LB and TSB media, Difco), microaerophilic growth (GasPak EZ Container system, (BD) with Brucella-blood agar (Anaerobe Systems), Campylobacter-Wollinella agar (Anaerobe Systems), and 2216 agar). Minimal marine salts supplemented with 2 mM Na_2_S-9H_2_O (Sigma) or 5 mM Na_2_S_2_O_3_ (Sigma) was employed to test for chemoautotrophic growth. Catalase activity was assayed using 3% H_2_O_2_ and Gram staining performed according to manufacturer's protocol (BD Life Sciences). Cell morphology was observed on a Zeiss Axioskop 2 (Carl Zeiss MicroImaging Inc.) after staining cells from early stationary phase cultures (2 days) with 4′,6-diamidino-2-phenylindole (DAPI, Sigma). Motility was scored as directional swimming in live cultures observed via light microscopy. Colony morphology was described after growth on 2216 agar at 28°C for 2 days (unless otherwise specified). Tests for tolerance of pH were conducted in 2216 broth pH-adjusted and buffered by the addition of acetic acid (range of pH4-5), NaH_2_PO_4_ (pH6-8), or Tris base (pH9-10) at 28°C and scored by culture turbidity after 2 days. Salinity tests were conducted in LB media omitting or adding NaCl and scored by culture turbidity. Temperature tolerance from 4 to 45°C was measured by evidence of colony growth on 2216 agar after 4 days. Tests for antibiotic sensitivity were conducted in 2216 marine broth supplemented with Nalidixic acid (4 mg L^−1^), Chloramphenicol (10 mg L^−1^), Ampicillin (100 mg L^−1^), Kanamycin (100 mg L^−1^), or Streptomycin (100 mg L^−1^).

### Genome sequencing, assembly, annotation, and analysis

Whole genome libraries were prepared (after Penn et al., 2014) for sequencing using the Illumina Genome Analyzer (Illumina, Inc.). Briefly, 5 μg of genomic DNA from each strain was sheared using Adaptive Focused Acoustic technology (Covaris, Inc.) to generate fragments 100–300 base pair (bp) in length. Fragments were blunt-ended, A-tailed and ligated with T nucleotide overhang Illumina forked paired end-sequencing adapters (Illumina, Inc.) containing bar codes for multiplex sequencing. Resulting libraries were size selected on an agarose gel to obtain 250 bp libraries. Libraries were then PCR amplified for 15 cycles based on determination of optimum number of cycles using qPCR. Libraries were multiplexed and sequenced to a targeted depth of 50X. Bacterial genomes were assembled into contigs using CLC Genomics Workbench 4 (Aarhus, Denmark). Contigs produced by the CLC assembly were uploaded to the Rapid Annotations using Subsystems Technology (RAST) server for identification and annotation of open reading frames (ORFs) (Aziz et al., 2008). Genomes corresponding to strain names are public in the RAST database. ORFs were also annotated by assignment to orthologous groups in the eggNOG Database (v3.0) (Powell et al., 2012) based on similarity searches with BLASTP (Altschul et al., 1997) with a threshold *e*-value < 1e^−20^ and where the aligned portion includes the predicted functional residues of the protein (as designated in the COG/NOG database).

To test if isolates from distinct phylogenetic lineages shared similar regions of DNA including phage or prophage elements, suggesting potential horizontal gene transfer within the holobiont, (1) isolate genome ORFs were compared to each other by BLASTN (minimum match identity = 95%), (2) the predicted proteome of each isolate was compared with BLASTP to the PHAST phage and prophage database (Zhou et al., 2011) with an *e*-value < 1e^−10^ followed by manual inspection of top matches, and (3) the nucleotide sequences were compared to all virus and phage genomes in the non-redundant nucleotide database (July 2014) by BLASTN. (4) To assess the potential for horizontal gene transfer between *N. vectensis* and the isolates, a BLASTN was performed between the contigs of all 10 assembled *N. vectensis* associate genomes and the scaffolds of the current *N. vectensis* genome (v.1, Putnam et al., 2007). Sequence matches were determined using an expected value cutoff of 1e^−30^. *N. vectensis* scaffolds containing bacteria-like DNA were manually inspected and analyzed with custom python scripts in order to determine their GC content, ambiguous base composition and size, which were used to assess the likelihood of horizontal gene transfer. Finally, to screen for factors of host-association protein sequences from annotated ORFs were compared a database of virulence factors (Chen et al., 2012) by BLASTP with an *e*-value < 1e^−10^ followed by manual inspection of top matches.

### Analysis of expressed RNA in field-collected and laboratory-acclimated anemones

#### Characterization of expressed 16S rRNA in *N. vectensis* from sippewissett marsh

One gram of *N. vectensis* polyps collected from Sippewissett Marsh July 2009 were preserved onsite in RNAlater and later homogenized in the TriPure Isolation Reagent (Roche Applied Science) with a mortar and pestle according to manufacturer's protocol. The homogenate was then mixed with chloroform at room temperature and centrifuged at 12,000 g for 30 min at 4°C. The upper aqueous phase containing RNA was mixed with 2.5 ml isopropyl alcohol and incubated at −80°C for 15 min, and then centrifuged at 12,000 rpm for 15 min at 4°C. The resulting RNA pellet was washed with 75% ethanol, air-dried and resuspended in DEPC treated nuclease free water and stored at −80°C. RNA was further purified prior to analysis as previously described (Sambrook and Russell, 2001). Briefly, RNA and phenol:chloroform:isoamylalcohol (25:24:1) were mixed at 1:1 ratio, vortexed for 15 sec and centrifuged at 14,000 g for 5 min. The aqueous phase was mixed with 0.1 volumes of ammonium acetate (pH 5.2) and 2.5 volumes of ice-cold 100% ethanol. Samples were gently mixed and incubated at −80°C for 30 min, and then centrifuged at 14,000 g for 30 min. Pellet was washed in 70% ethanol and resuspended in DEPC treated nuclease free water and stored at −80°C. One microgram of purified total RNA was used for cDNA synthesis. The first strand cDNA synthesis was carried out using Transcriptor First strand cDNA synthesis kit (Roche) according to manufacturer's protocol, with the exception that pentadecamer primers (5′-NNNNNNNNNNNNNNN-3′) synthesized by Integrated DNA Technologies (Coralville) were used for random amplification of total RNA instead of the random hexamers supplied with the kit (Stangegaard et al., 2006). The cDNA was used in place of genomic DNA as a template for 16S rRNA gene amplification and cloning as described above.

#### Characterization of expressed bacterial ORFs in laboratory-raised *N. vectensis* through a pilot-scale metatranscriptome study

Laboratory-raised *N. vectensis* adults were incubated and treated to reduced microbial contamination, as described earlier. Twenty *N. vectensis* polyps (approximately 2 cm each) were homogenized in TRIzol reagent (Life Technologies) and their RNA was extracted according to manufacturer's instructions, including treatment with DNAse followed by phenol-chloroform-extraction. The RNA was divided between six samples that were each subjected to various combinations of rRNA depletion protocols as an initial screen of protocol effectiveness. These depletion methods were designed to enrich for microbial mRNAs and eliminate eukaryotic RNAs and bacterial rRNAs and are summarized in **Table 5**. Unprocessed total RNA was included as a reference sample. All RNA samples were transcribed to cDNA (SuperScript Kit Catalog # 11917-020) following treatments for depletion of rRNA and eukaryotic RNA. To prepare the Illumina libraries, cDNA for each sample was sheared to fragments of between 100 and 300 base-pairs, purified, ligated into proprietary Illumina Adaptor sequences (Illumina, Inc., San Diego, CA) with unique 6 base-pair barcode sequences to designate samples for multiplexing within a single lane. Barcoded adaptor-ligated cDNA was then subject to size selection to remove self-ligated adaptors. Cleaned and merged adaptor-ligated cDNA was sequenced using the Illumina-GAII platform (as described in Penn et al., 2014). The cDNA was sequenced as paired end reads (100 bp × 2) on an Illumina GA-II. The resulting FastQ file was sorted by unique barcode then sequences were truncated by removing the barcodes, the Illumina adaptors and tandem repeat sequences were removed utilizing perl and python scripts. Sequence pairs were then compared against the Silva large and small subunit rRNA databases (Quast et al., 2013) using BLASTN. The pairs having one or both ends matching a ribosomal RNA database sequence with a bitscore > 50.0 were removed. Remaining sequences were compared against a custom database of bacterial and *N. vectensis* 5S rRNA and ITS sequences using BLASTN, and again, those pairs having one or both ends matching a sequence within one of these databases with bit score > 50.0 were removed. Following rRNA separation, remaining paired sequences that had overlapping sequence were merged using the software program SHERA with confidence metric ≥ 0.7 (Rodrigue et al., 2010).

#### Annotation of assembled and individual metatranscriptome sequences from laboratory-raised N. vectensis

Putative mRNA sequences were assembled in CLC genomics workbench with the following settings (mismatch cost = 2; Insertion cost = 3; Deletion cost = 3; Length fraction = 0.5; Similarity fraction = 0.8). The contigs were then used for BLASTX search against the NR database using a low complexity filter and the top 100 hits kept. These results were loaded into MEGAN to identify the taxonomic matches of the contigs using the lowest common ancestor (LCA) method. LCA parameters for taxonomic assignments in MEGAN were set with a minimum support of 10, a minimum bit score of 50, maximum e-value of 0.01, only considering matches that lie within the top 10% of the best score for a particular sequence, and the minimum complexity filter was set at 0.44. Putative mRNAs was also compared against all sequences in the NCBI database using BLASTX (Altschul et al., 1997) with parameters (-m 8 -W 3 -e 20 -Q 11 -F “m S”). The BLASTX results were imported into MEGAN with bit score cutoff 40.0 and the lowest common ancestor cutoff being 2 matches (Huson et al., 2007). Unmerged sequence pairs with ends matching different domains (Bacteria, Archaea, and Eukarya) were discarded; those matching the same domain were annotated with more specific taxonomy.

#### Mapping the *N. vectensis* metatranscriptome to *N. vectensis* bacterial isolate genomes

Non-ribosomal reads were merged into one Fasta file and imported into CLC Genomics Workbench (CLC Bio, Cambridge, MA) as unpaired sequences. The sequences were aligned with the annotated isolate reference genomes using the “map reads to reference sequence” function of CLC with parameters adjusted to provide stringent mapping of short cDNA sequences (Similarity = 0.9; Length Fraction = 0.5). Mapping results were manually inspected for coverage and sequence identity. ORFs from bacterial isolates with greater than 200 bp of consensus sequence coverage from mapped reads with >95% identity were considered for further analysis.

## Results

### Molecular diversity of microbiota associated with *N. vectensis* at three salt marshes

**Figure 2:**
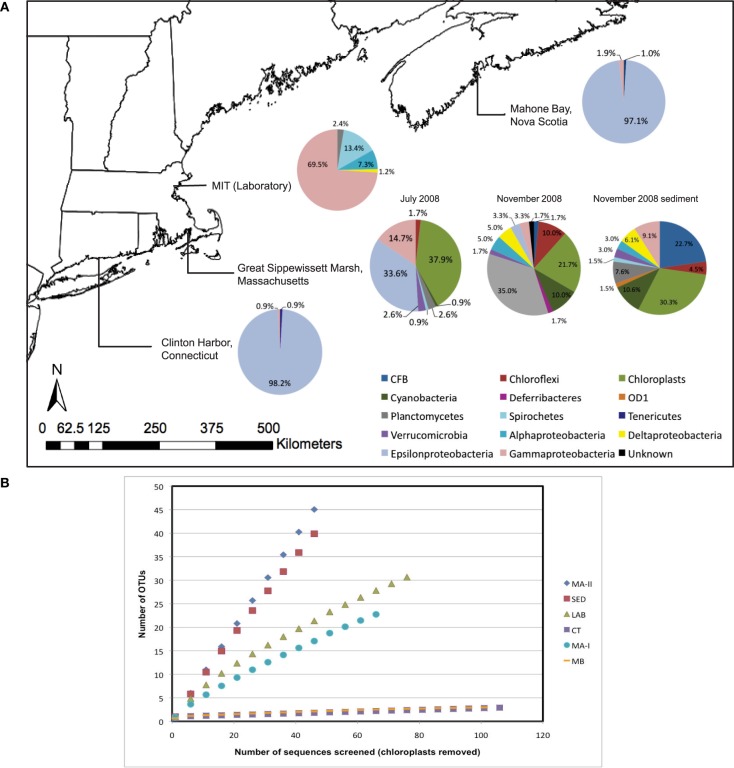
**(A)** Map of sampling locations and distributions of microbial diversity in field and laboratory-acclimated anemones and sediment clone libraries. **(B)** Rarefaction curves for bacterial 16S rRNA OTUs (excluding chloroplasts) obtained from clone libraries prepared from laboratory-reared (LAB) and field-collected *N. vectensis* from Sippewissett in June 2008 (MA-I) and November 2008 (MA-II), Clinton (CT) and Mahone Bay (MB), and with Sippewissett sediment in November 2008.

**Figure 3:**
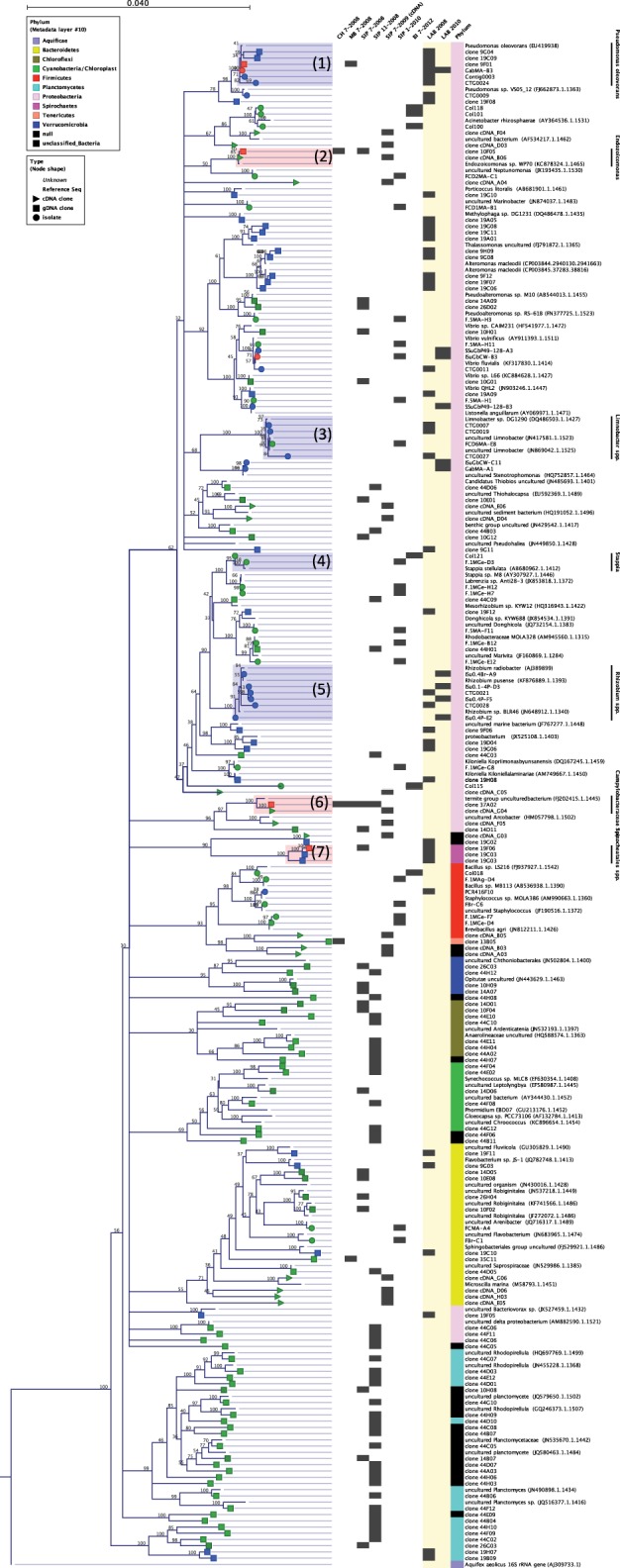
**Neighbor-joining phylogeny of 16S rRNA gene sequences from clones and isolates analyzed in this study with the most closely related reference sequences from the Silva database with >95% rRNA identity**. Symbols at branch termini denote origin of sequence from isolate (circle), cloned 16S rRNA from gDNA (square) or from cDNA (triangle). Symbol colors correspond to sample origin: Salt marsh collected anemones (green), laboratory-acclimated anemones (blue) or from both field and lab-acclimated anemones (red). Chart to the right of tree specifies the specific sample and date of sequence origin. Phylum is indicated at the far right and corresponds to the legend on the figure. Highlighted sequence clusters correspond to taxa discussed in this study (1) *Pseudomonas oleovorans*, (2) *Endozoicomonas* spp., (3) *Limnobacter* spp., (4) *Stappia* spp., (5) *Rhizobium radiobacter*, (6) uncultured Campylobacter lineage (note: the origin of the “termite group” sequence FJ202415 is the coral *Orbicella (Montastrea) faveolata*), (7) uncultured OTUs with highest sequence identity to a coral-derived Spirochete.

A total of 393 non-chimeric Bacterial and chloroplast 16S rRNA gene sequences (*E. coli* positions 27–805) were obtained from *N. vectensis* from Mahone Bay, Nova Scotia (MB), Clinton Harbor, Connecticut (CT) and Sippewissett Marsh, Massachusetts (MA-I, MA-II) (Figure 2A, Table 1). An additional 39 16S rRNA gene sequences were recovered from cDNA libraries prepared from *N. vectensis* total RNA (Sippewissett Marsh, MA-IV), 82 Bacterial 16S rRNA genes were recovered from laboratory-reared *N. vectensis* and 66 16S rRNA sequences were obtained from Sippewissett Marsh surface sediments (Table 1). Archaeal 16S rRNA genes were not recovered by amplification of *N. vectensis* DNA (20 ng) with the Archaeal primer pair 21F to 958R. Three to ten bacterial phyla were recovered from samples of *N. vectensis* consisting of representatives from Cytophaga-Flexibacter-Bacteroides (CFB), Chloroflexi, Cyanobacteria, Deferribacteres, Firmicutes, OD1, Planctomycetes, Proteobacteria, Spirochetes, Tenericutes and Verrucomicrobia (Figures 2A, 3). Operational taxonomic units (OTUs) were defined as clusters of 16S rRNA sequences sharing >99% identity (i.e., a ribotype).

Bacterial sequences associated with *N. vectensis* from the MB and CT salt marshes were dominated by a single ε-Proteobacterial OTU in the Order Campylobacterales which corresponded to 98 and 97% of the MB and CT clone libraries, respectively; 34 and 3% of sequences from *N. vectensis* collected from Sippewissett Marsh July 2008 and November 2008, respectively (Figure 2A) and 26% of the bacterial cDNA clones, indicating expressed rRNAs from Sippewissett Marsh *N. vectensis*, in July 2009 (Figure 3, cluster 6). This Campylobacterales ribotype appears to be part of an uncultured lineage sharing 96% identity with clones from *Orbicella faveolata* (Sunagawa et al., 2009) (FJ202415 in Figure 3, cluster 6) and sharing ≤85% identity with the closest cultured relatives in the bacterial genera *Helicobacter, Arcobacter*, and *Sulfurovum lithotrophicum* representing gastrointestinal pathogens (Engberg et al., 2000) as well as sulfur-oxidizing chemoautotrophs (Inagaki et al., 2004).

Two additional OTUs were distributed in multiple clone libraries from field-collected *N. vectensis*. An OTU with 99.6% 16S rRNA identity to the marine-invertebrate endobiont *Endozoicomonas elysicola* (Schuett et al., 2007) was associated with anemones from Clinton Harbor, CT and Sippewissett Marsh, MA, representing 0.9 and 4.9% of cloned sequences from 16S rRNA gene libraries, respectively, and 23% of bacterial cDNA clones representing expressed rRNA from Sippewissett Marsh *N. vectensis* (Figure 3, cluster 2). In addition, an OTU sharing 99.6% rRNA identity with isolates of *Pseudomonas pseudoalcaligenes* (now reclassified as *P. oleovorans*, Saha et al., 2010) a widely distributed environmental bacteria and an opportunistic pathogen (Gilardi, 1972; Yamamoto et al., 2000), were recovered from anemones from Mahone Bay (Figures 2A, 3) representing 1.9% of cloned sequences.

*N. vectensis* from MB and CT revealed a surprisingly low diversity of associated bacterial types (chao1 3.5 and 4, respectively) dominated by the Campylobacterales OTU while anemones from Sippewissett Marsh harbored a higher diversity (chao1 78 and 1059). There is no obvious explanation for the low diversity of microbial sequences from anemones from Clinton Harbor, CT and Mahone Bay, NS relative to anemones from Sippewissett Marsh, MA. Comparison of sequence types associated with Sippewissett Marsh sediments collected in November 2008 (chao1 283) with sequences recovered from the anemones collected at the same location and time (November 2008) reveals a similar distribution of Cyanobacterial and chloroplast sequences (Figure 2A), suggesting some sequence richness in anemones from Sippewissett Marsh may be due to a contribution of sediment associated-bacteria. Moreover, a high proportion of sequences (23%) associated with Sippewissett Marsh *N. vectensis* (November 2008) were from diatom chloroplasts. We have observed diatoms attached to the external body wall of *N. vectensis* (Figures 1C,D). Comparisons between anemones and sediment clone libraries cannot be made with the CT and MB samples as the sediments in these locations were not collected. Notably, the previously described OTUs associated with *N. vectensis* at multiple salt marshes (i.e., sequences similar to Campylobacterales, *Endozoicomonas elysicola*, or *Pseudomonas oleovorans*) were not observed in Sippewissett Marsh sediments, although rarefaction analysis indicated that the sediment clone library diversity was not sampled to saturation (Figure 2B).

### Molecular diversity of microbiota associated with laboratory-reared *N. vectensis*

Laboratory-raised *N. vectensis* were associated with a similar magnitude of bacterial diversity as anemones collected from the field (chao1 80; Table 1) however, the bacterial community composition was notably different. Laboratory-reared anemones were associated with a majority of γ-Proteobacterial sequences (75%), in contrast to field-collected anemones that were associated with ≤16% γ-Proteobacterial sequences (Figure 2A). Only two microbial OTUs observed in wild anemones were also recovered from the laboratory-reared *N. vectensis*; *Pseudomonas oleovorans* and a novel Spirochete OTU (92.8% 16S rRNA identity with an uncultured clone from a deep-sea coral) that was also recovered from the Sippewissett Marsh sediment clone library.

### Species-specific PCR of *N. vectensis* microbial associates

**Figure 4:**
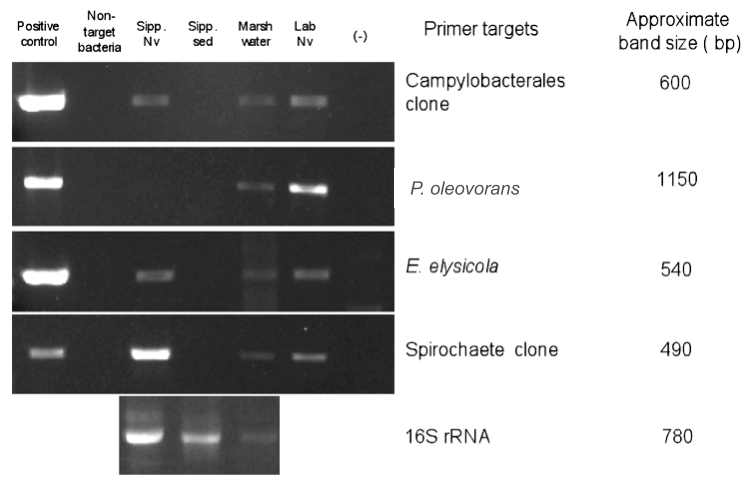
**Species-specific PCR based detection of microbial populations in genomic DNA extracted from samples collected in June 2009 from Sippewissett Marsh or the laboratory-acclimated ***N. vectensis*** population**. Lanes 1–6 correspond to (1) cloned 16S rRNA sequence as positive control, (2) negative control consisting of non-target 16S rRNA DNA, (3) *N. vectensis* from Sippewissett Marsh, (4) Sippewissett Marsh sediment, (5) Seawater from Sippewissett Marsh at sampling site, (6) laboratory-adapted anemones, and (7) No template control (master mix + water only), respectively. Positive controls were 10^5^ copies of target 16S rRNA and samples were 10 ng DNA.

To determine whether the four OTU's associated with *N. vectensis* in multiple clone libraries during Summer/Fall 2008 (i.e., the Campylobacterales and Spirochete OTUs, and the *Endozoicomonas elysicola*-, and *Pseudomonas oleovorans*-like OTUs) remained associated with both laboratory-reared and Sippewissett Marsh *N. vectensis* collected in June 2009 (representing a timespan of 9–12 months) as well as to screen for their presence in the surrounding marsh habitat (sediment and water), specific PCR assays were designed for each OTU. These analyses confirmed that the Campylobacterales and Spirochete OTUs and *Endozoicomonas elysicola* remained associated with both laboratory-reared and field-collected anemones in June 2009 (Figure 4), however *Pseudomonas oleovorans* amplicons were not recovered from the field-collected anemone DNA. Marsh water DNA yielded the expected sized amplicons for all four-sequence types, although PCR inhibition of amplification was apparent by the reproducible faint band intensity of 16S rRNA amplicon from universal eubacterial primers, relative to other environments. In contrast, surface sediments did not appear to be associated with any of these four OTUs, and a positive signal for the universal 16S rRNA amplicon indicated PCR inhibition was not a confounding factor in the sediment analysis (Figure 4). Non-detection of Campylobacterales, *Endozoicomonas elysicola*, and *Pseudomonas oleovorans* amplicons in the sediment sample (June 2009) is consistent with their absence from the clone library prepared from surface sediments collected in November 2008 (data not shown). This suggests that the association of these ribotypes with *N. vectensi*s may be more specific than ingestion of detritus or attachment of the surrounding sediment to the anemone surface. However, the absence of a Spirochete OTU-specific PCR amplicon from marsh sediment was surprising, and may be due to a low concentration of this population's DNA in the sediment in June 2009, in contrast to November 2008 when a single sequence was observed in the sediment clone library.

### Diversity of isolates cultured from *N. vectensis*

Seven different media combinations were used to isolate a total of 132 bacterial strains from the field anemones and 511 bacterial strains from anemones maintained in the lab (Table 2). These strains were classified by 16S rRNA RFLP and sequencing. These strains corresponded to a total of 19 different ribotypes, among which 17 ribotypes were recovered from the field and 5 ribotypes from the lab (Table 2). Types recovered from multiple samples from the field generally did not overlap, while similar types (dominated by *Pseudomonas oleovorans* and *Rhizobium radiobacter* were recovered from all laboratory samples on all media formulations. There was little overlap between populations recovered from lab vs. field—notable exceptions were a *V. furnissii*-like ribotype. Most isolates from field collected anemones shared >95% nucleotide similarity with isolates or cloned sequences obtained from other anthozoans (primarily stony corals), suggesting potentially conserved mechanisms for association with anthozoans. Three ribotypes observed in the 2010 culture collection matched ribotypes recovered from an earlier survey of culturable diversity associated with laboratory-raised anemones (in 2008 and 2009) where isolates were recovered from on 2216 media. Quantitative data on strain distribution from this 2008 study are not available, however isolates of *P. oleovorans, Limnobater thiooxidans*, and *R. radiobacter* were archived during this study and serve as a reference for strains isolated in 2010. Bacterial isolates observed in multiple samples or with similarity to associates of other Anthozoan hosts were selected for additional physiological and genomic characterization. These included *Pseudomonas oleovorans* isolated from laboratory-acclimated *N. vectensis* in 2008 (Po-B4) and 2010 (Po-Gab and Po-Is) and strain Po47 from anemones donated from John Finnerty's laboratory (Boston University, 2010) (Figure 3, cluster 1), *R. radiobacter* isolated from lab-acclimated anemones in 2008 (Rr-D5 and Rr-D8) and 2010 (Rr-Is) (Figure 3, cluster 5). In addition, two *Limnobacter* isolates from salt marsh-collected *N. vectensis* in 2010 (Lt-F1 and Lt-FCMA) matched sequences from isolates of laboratory-acclimated anemones obtained in 2008 (Figure 3, cluster 3) and a single *Stappia* isolate was selected that matched a cloned sequence from a stony coral (Ss-F1) and sequences from isolates that were subsequently recovered from anemones collected at Belle Island Marsh, near Boston MA in 2012 (Figure 3, cluster 4). Isolates from the genus *Vibrio* (in particular *V. furnissii*) were excluded from this analysis because of their ubiquitous recovery from coastal environments and high coverage of this particular genus and species in characterized culture collections and among genome-sequence repositories. Notably, despite varied cultivation methods (including targeting aerobic, anaerobic and microaerophilic growth) no isolates of the Campylobacter, *Endozoicomonas*, or Spirochete OTUs associated with multiple anemone samples in the cultivation-independent characterization were recovered during the isolations.

**Table 2:** **Summary of bacterial isolates recovered from ***Nematostella vectensis*** (Nv) maintained in the laboratory or collected from Sippewissett Marsh (March 2010)**.

**Ribotype**	**Highest similarity to:**	**From lab Nv**	**From field Nv**
1	*Rhizobium radiobacter* (98%)	13.1% (67/511)	0.0% (0/132)
2	*Arenibacter troitsensis* (99%)	0% (0/511)	6.8% (9/132)
3	*Bacillus hwajinpoensis* (99%)	0% (0/511)	36.1% (48/132)
4	*Brevibacillus agri* (99%)	0% (0/511)	4.5% (6/132)
5	*Donghicola eburneus* (99%)	0% (0/511)	0.8% (1/132)
6	*Flavobacterium gelidilacus* (98%)	0% (0/511)	3% (4/132)
7	*Kiloniella laminariae* (98%)	0% (0/511)	0.8% (1/132)
8	*Labrenzia alba* (98%)	0% (0/511)	1.5% (2/132)
9	*Limnobacter thiooxidans* (99%)	0% (0/511)	14.3% (19/132)
10	*Listonella anguillarum* (99%)	0.2% (1/511)	0.8% (1/132)
11	*Marinobacter flavimaris* (99%)	0% (0/511)	1.5% (2/132)
12	*Marivita cryptomonadis* (98%)	0% (0/511)	5.3% (7/132)
13	*Neptunomonas japonica* (95%)	0% (0/511)	0.8% (1/132)
14	*Pseudoalteromonas prydzensis* (98%)	0% (0/511)	0.8% (1/132)
15	*Pseudomonas oleovorans* (98%)	35.4% (181/511)	0% (0/132)
16	*Staphylococcus hominis* (99%)	0% (0/511)	4.5% (6/132)
17	*Stappia stellulata* (98%)	0% (0/511)	6% (8/132)
18	*Stenotrophomonas maltophila* (97%)	47.4% (242/511)	0% (0/132)
19	*Vibrio furnissii* (99%)	3.9% (20/511)	12% (16/132)

Ten bacterial strains isolated from *N. vectensis* were subjected to a suite of physiological tests to characterize their optimal growth conditions (Table 3). All of the isolates grew on heterotrophic media at 28°C under aerobic conditions with colonies evident after 24–48 h (*Pseudomonas, Rhizobium, Stappia*) or 72 h (*Limnobacter*). All strains grew over the pH range of 6–10 with optimal growth at pH 7–8, were catalase positive and gram(–), did not exhibit hemolysis on blood agar plates and exhibited a range of heterotrophic growth under microaerophilic conditions. All strains exhibited directional motility when observed by light microscopy. None of the strains were observed to grow chemoautotrophically with NaS or NaS_2_O_3_ as an electron donor after 1 week. The *Pseudomonas oleovorans* cells were 0.5 × 1–1.5 μm rods forming 0.5 to 1 mm diameter colonies on 2216 media with variable opacity and texture after 2 days (Table 3). Growth was observed from 16 to 45°C with optimal growth at 28 and 37°C. Salinity tolerance ranged from 0 to 5% (optimal 2–3%). *Pseudomonas* strains revealed resistance to multiple tested antibiotics (Nalidixic acid, Chloramphenicol and Ampicillin). *R. radiobacter* isolates had variable cell size, short and stout rods (0.5–0.7 μm × 0.7–1 μm) to slender rods (0.7 × 2–2.5 μm), and formed punctate opaque colonies after 2 days on 2216 media. Growth was observed from 16 to 37°C with optimal growth at 28°C and variable growth at 45°C. Salinity tolerance varied by strain with all strains growing from 0 to 3% (optimal 2–3%) and strain Rr-D5 tolerating up to 7% salinity. All *Rhizobium* strains were resistant to Streptomycin and exhibited variable resistance to other tested antibiotics with strain Rr-D5 exhibiting resistance to all 5 antibiotics tested. *Limnobacter thiooxidans* strains were motile rods (0.5 × 1–1.5 μm) and formed 0.5–1 mm translucent colonies with variable texture after 72 h. Growth after 4 days occurred from 22 to 37°C with variable growth at 16°C. The optimal salinity for growth was 2% and strains varied in salinity tolerance (1–2% for strain Lt-F1 and 0–3% for strain Lt-FCMA). *Limnobacter thiooxidans* strains were sensitive to all five antibiotics tested. The *Stappia stellulata* isolate formed motile rods (0.5 − 1.5–2 μm) and grew from 16 to 45°C (optimal 22-37°C) and at salinities from 2 to 5% (optimal 3%). This strain was resistant to Nalidixic acid and Streptomycin.

**Table 3:** **Physiological characterization of ***N. vectensis*** associated strains selected for genome-sequencing**.

**Strain ID**	**Medium preference**												**Growth condition**									
		**Aerobic growth (1)**			**Microaerophilic growth (2)**					**(3)**			**Temperature (5)**						**Salinity (6)**	**pH (7)**		
**16SrRNA top BlastN**	**Isolation Date/Origin**	**LB**	**TSB**	**2216**	**Na_2_S**	**Na_2_S_2_O_3_**	**2216**	**C-W**	**Bruc. blood**	**AB resistance**	**Cell morphology (1)**	**Colony morphology (4)**	**4°C**	**16°C**	**22°C**	**28°C**	**37°C**	**45°C**			**Catalase**	**Gram Staining**
Po-Gab—P. oleovorans subsp. oleovorans (98.2%)	Isolated from lab anemones (2/2010). Gentamycin treatment after tissue homogenization; strain was isolated on 2216 marine agar	+++	+++	+	−	−	+	+++	+++	NAR, CmR, ApR	Motile rod 1~1.5 × 0.5 μm	1 mm, ivory, opaque, circular, entire margin, bumpy surface	−	+	++	+++	+++	+	0–5% (max. 2–3%)	pH6–10 (max. pH7)	++	−
Po-ls—P. oleovorans subsp. oleovorans (98.2%)	Isolated from lab anemones (2/2010). Enriched in sulfide-gradient-tube with ASW and 0.1 mM NaN03; isolation on 2216 marine agar	+++	+++	+	−	−	+	+++	+++	NAR, CmR, ApR	Motile rod 1~1.5 × 0.5 μm	1 mm, ivory, opaque, circular, entire margin, bumpy surface	−	+	++	+++	+++	+	0–5% (max. 2–3%)	pH6–10 (max. pH7)	++	−
Po-B4—P. oleovorans subsp. oleovorans (98.2%)	Isolated from lab anemones (1/2008). Homogenate plated on 2216 marine agar	+++	+++	+	−	−	+	+++	+++	NAR, CmR, ApR	Motile rod 1~1.5 × 0.5 μm	1 mm, translucent, ivory, circular bumpy surface, entire margin	−	+	++	+++	+++	+	0–5% (max. 2–3%)	pH6–10 (max. pH7)	++	–
Po-47—P. oleovorans subsp. oleovorans) (98.2%)	Isolated on 2216 marine agar from homogenized laboratory-raised anemones obtained from John Finnerty's Lab (BU) (11/2009)	+++	+++	+	−	−	+	+++	+++	NAR, CmR	Motile rod 1~1.5 × 0.5 μm	0.5–0.7 mm, translucent, ivory, circular with entire margin, smooth shiny surface	−	+	++	+++	+++	+	0–5% (max. 2–3%)	pH6–10 (max. pH7)	++	−
Lt-F1—Limnobacter thiooxidans (98.9%)	Field animals were used as innocula (4/2010); isolated on 0.1X strength 2216 marine broth solidified with gellan gum	+, 72 h	−, 72 h	+, 72 h	−	−	+	−	+	None observed	Motile rod 1~1.5 × 0.5 μm	0.5 mm ivory, translucent, circular entire margine, smooth and shiny surface	−	+	+	+	+	−	1–2% (max. 2%)	pH6–10 (max. pH8)	++	−
Lt-FCMA—Limnobacter thiooxidans (98.9%)	Field animals were used as innocula (4/2010); Collagenase was used for tissue maceration; isolated on 2216 marine agar	++, 72 h	−, 72 h	+, 72 h	−	−	+	−	+	None observed	Motile rod 1~1.5 × 0.5 μm	1 mm ivory, translucent/transparent, circular, umbonate, shiny/smooth surface	−	−	+	++	++	−	0–3% (max. 2%)	pH6–10 (max. PH7)	++	–
Rr-ls—Rhizobium radiobacter (97.7%)	Lab anemone inocula (2/2010); Enriched in sulfide-gradient-tube with ASW and 0.4mM NaN03; further isolation on Brucella Blood agar	+++	+++	+	−	−	+	+++	++	NAR, SmR, CmR, KnR	Short and stout rods 1.5 × 0.7 μm, relatively less motile, clusters of 10–20 cells.	Pinpoint, ivory, opaque, circular smooth with entire margin	−	+	++	+++	+	−	0–3% (max. 2%)	pH6–10 (max. pH7)	++	−
Rr-D8—Rhizobium radiobacter (97.7%)	Isolated on 2216 marine agar from homogenized laboratory-raised anemones (1/2008)	+++	+++	++	−	−	++	+++	++	SmR	Slender rods 2–2.5 × 0.7 μm, relatively less motile and occur in pairs	Pinpoint, ivory, opaque, circular smooth surface, entire margin	−	+	++	+++	+	+	0–3% (max. 2%)	pH6–10 (max. pH7)	++	−
Rr-D5—Rhizobium radiobacter (97.7%)	Isolated on 2216 marine agar from homogenized laboratory-raised anemones (1/2008)	+++	+++	++	−	−	+	+++	+++	NAR, SmR, CmR, KnR, ApR	Short and stout, motile rods 0.7–1 × 0.5–0.7 μm in size	Pinpoint, ivory, opaque, circular smooth surface, entire margin	−	+	++	+++	+++	+	0–7% (max. 2–3%)	pH6–10 (max. PH7)	++	−
Ss-F1—Stappia stellulata (98.7%)	Field animals were used as innocula (4/2010); isolated on 0.1X strength Marine (= 2216) broth solidified with gellan gum	+++	+++	++	−	−	++	+++	+++	NAR, SmR	Motile rod 1.5–2 × 0.5 μm	Pinpoint, ivory, opaque, circular smooth surface with entire margin	−−	++	+++	+++	+++	+	2–5% (max. 3%)	pH6–10 (max. PH7)	++	−

*+++, very good growth; ++, good growth; +, weak growth; –, no growth. (1) Growth under aerobic conditions: All strains were shaken at 28°C for 48 h unless otherwise specified. (2) Growth under Microaerophilic conditions. All strains were incubated with GasPak EZ Anaerobic Pouch System (BD) at 28°C for 3 day; MM, Minimal Media; C-W, Campylobacter Wollinella Agar; Bruc. Blood, Brucella Blood Agar. (3) Antibiotic Resistance. R, Resistant; NA, 4 ppm Nalidixic acid; Sm, 100 ppm Streptomycin; Cm, 10 ppm Chloramphenicol; Kn, 100 ppm Kanamycin; and Ap, 100 ppm Ampicillin. (4) Colony morphology determined after growth on 2216 agar at 28°C for 2 days unless otherwise specified (5) All strains were grown on 2216 plate and observed after 4 days. (6) All strains were grown on LB broth omitting NaCl at 28°C for 3 days. (7) All strains were grown on 2216 broth buffered with acetic acid, NaH2PO4, or Tris base at 28°C for 48 h*.

### Characterization of genomes from bacterial isolates

**Figure 5:**
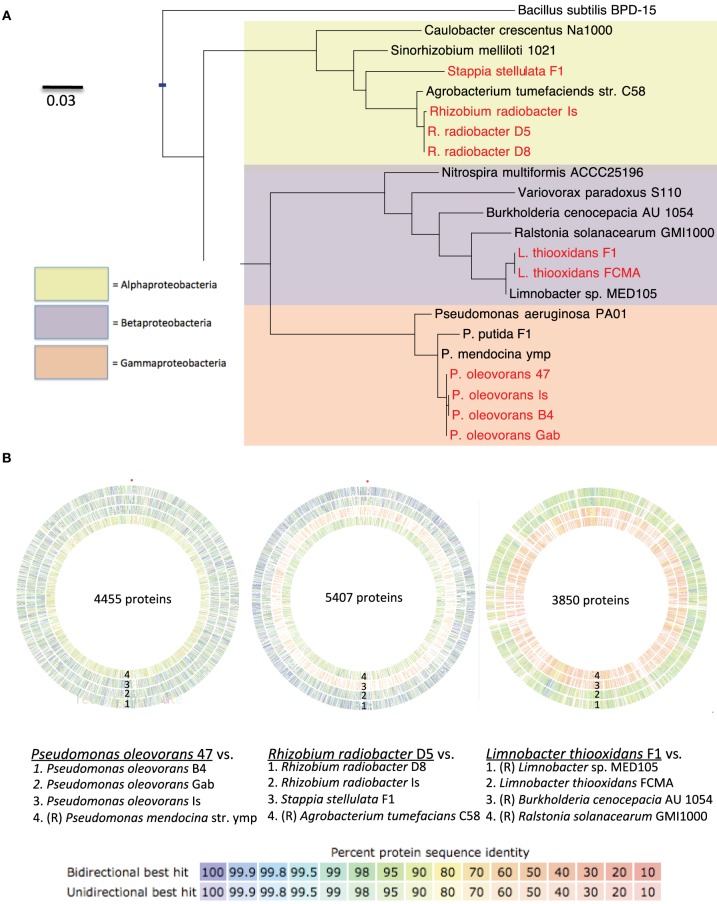
**(A)** Maximum-likelihood phylogenetic tree of the 16S rRNA gene from cultured isolates from *N. vectensis* (red) and reference strains (black). Scale bar is average substitutions per site. **(B)** Comparison of genome sequences from the most closely related publically available reference strains (R) to *N. vectensis* holobiont isolates. Genome assemblies from isolates and reference strains were imported into RAST and annotated via its built in ORF finder function. Concentric circles represent comparison of the partial assemblies via pairwise comparison of ORFs from (from left to right) *Pseudomonas* strains, Rhizobial strains and Beta-proteobacterial strains with other strains isolated from *N. vectensis* or closely related reference genomes [Outer to inner rings, as labeled in figure, (R) denotes genome obtained from the RefSeq database]. For the *Pseudomonas* group, the plots depict pairwise comparison of strain Po-47 to each of the following four genomes (Po-B4, Po-Gab, Po-Is and *P. mendocina* strain ymp). For the Rhizobial genomes the plots depict pairwise comparison of strain Rr-D5 to each of the following four genomes [Rr-D8, Rr-Is, Ss-F1 and *Agrobacterium tumefaciens* (updated scientific name: *Rhizobium radiobacter*)]. For the Betaproteobacterial genomes the plots depict pairwise comparison of strain Lt-F1 to each of the following four genomes [*Limnobacter* spp. MED105, Strain Lt-FCMA (this study), *Burkholderia cenocepacia* AU1054, and *Ralstonia solanacearum* GMI1000]. Colored bars stacked to comprise the concentric rings represent shared ORFs (determined by bi- and uni- directional BLAST analysis) and the color represents the average protein sequence similarity between orthologs with the color scale representing the range of this value.

Estimated sequence coverage for isolate genomes ranged from 15.5x - 82.1x although none of the genomes could be closed (Table 4, Figure 5). Annotation of genomes with the RAST pipeline revealed multiple pathways for utilization of carbohydrates and proteins, consistent with observed heterotrophic growth on complex media. To identify potential mechanisms for host-association, genes with homology to virulence factors were identified through review of the RAST annotations and by homology to the virulence factor database VFDB (Chen et al., 2012). ORFs homologous to virulence factors commonly associated with both pathogenic and non-pathogenic Proteobacteria were observed in all isolates including genes mediating expression of flagellar motility and chemotaxis, general secretion (type II), type IV pili, fimbrae, iron transport, hemolysis (hlyA, B, D), siderophore biosynthesis, and superoxide dismutase (sodAB).

**Table 4:** **Assembly Statistics of Isolate Genome Sequences^a^**.

Strain name^*^	**Origin of *N. vectensis* host: date of isolation**	**No. of Post-QC Sequence Pairs (RPs); Adaptor Trimmed Sequences (ATRs); (estimated genome coverage)**	**Genome size in base Pairs (GC%)**	**No. of contigs, (N50) and largest contig (bp)**	**No. of annotated ORFs^b^**	**No. of unique NOG/COG^c^annotations**
Po_B4	Thompson Lab, MIT: Spring 2008	517,635 RPs 138,192 ATRs (15.5×)	5,410,491 (64.1%)	6720 (1075) 10,216	6781	2206
Po_Gab	Thompson Lab, MIT: 2/3/10	822,162 RPs 220,986 ATRs (25.3×)	5,196,558 (64.9%)	4429 (1695) 33,942	6372	2252
Po_Is	Thompson Lab, MIT: 2/8/10	629,033 RPs 164,238 ATRs (19.4×)	5,288,085 (64.6%)	5282 (1391) 9535	6553	2292
Po_47	Finnerty Lab, Boston Univ.: Fall 2009	608,695 RPs 150,484 ATRs (18.6×)	5,254,749 (64.6%)	5547 (1314) 13,599	6359	2203
Lt_F1	Marsh, Sippewissett, MA: 4/15/10	1567,436 RPs 222,958 ATRs (82.1×)	3,447,759 (51.7%)	203 (30,681) 93,380	3580	1666
Lt_FCMA	Marsh, Sippewissett, MA: 4/15/10	674,674 RPs 99,317 ATRs (38.3×)	3,212,319 (52.3%)	559 (10,292) 31,966	3726	1571
Rr_D5	Thompson Lab, MIT: 2/3/10	509,968 RPs 148,050 ATRs (15.8×)	5,376,246 (59.1%)	6285 (1151) 18,386	7685	2094
Rr_D8	Thompson Lab, MIT: Spring 2008	613,428 RPs 170,015 ATRs (18.4×)	5,488,699 (59.1%)	5624 (1395) 16,049	7543	2126
Rr_Is	Thompson Lab, MIT: 2008	681,511 RPs 203,168 ATRs (20.5×)	5,430,112 (59.3%)	4783 (1626) 10,588	7323	2141
Ss_F1	Marsh, Sippewissett MA: 4/15/10	717,642 RPs 231,719 ATRs (25.3×)	4,420,534 (65.3%)	3410 (1932) 13,551	5487	1756

* *Pseudomonas oleovorans (Po), Limnobacter thiooxidans (Lt), Rhizobium radiobacter (Rr), Stappia stellulata (Ss)*.

a *Genome assemblies were carried out in CLC Genomics Workbench version 4. Genome size, N50 and largest contig size are calculated by CLC Genomics Workbench. Genome coverage is calculated as the ratio of the bases of Illumina reads assembled in CLC (Mb) to the predicted genome size (Mb)*.

b *Open reading frames (ORFs) were identified and annotated via the Rapid Annotations using Subsystems Technology (RAST) server (Aziz et al., 2008)*.

c *ORFs were assigned to orthologous groups in the eggNOG Database (v3.0) (Powell et al., 2012) based on similarity searches with BLASTP (Altschul et al., 1997) with a threshold e-value < 1e^-20^ and where the aligned portion includes the predicted functional residues of the protein (as designated in the COG/NOG database)*.

*Pseudomonas oleovorans* genomes were most similar to sequenced genomes of *P. mendocina* strains ymp and NK-01 (Guo et al., 2011) (Figure 5). The predicted genome size was 5.20–5.41 Mb with 15.5–25.3x coverage and 64.1–64.9% GC content. The average nucleotide identity of orthologs shared between *Pseudomonas* isolates obtained from *N. vectensis* ranged from 94.91 to 97.08%. In addition to the virulence factors described above, BLASTX hits against the virulence factor database revealed hits against urease and one gene in the type VI secretion pathway (icmF).

*Rhizobium* genomes were most similar to sequenced strains of *Agrobacterium tumefacians* (revised name *R. radiobacter*) (Figure 5). The predicted genome sizes were 5.38–5.49 Mb with 15.8–20.5x coverage and 59.1–59.3% GC. The average nucleotide identity of orthologs shared among the isolates from *N. vectensis* ranged from 95.5 to 97.46%. In addition to the common Proteobacterial virulence factors identified above *Rhizobium* strains included proteins with significant similarity to ureases, type VI secretion proteins (vgrG and IcmF), antibiotic resistance proteins (including tetAB), and proteins involved in isochorismate and salicylate biosynthesis, which are linked to production of bioactive compounds.

*Limnobacter thiooxidans* genomes of strains were most similar to the partially assembled genome of *Limnobacter* strain MED105 and the completed genomes of the Betaproteobacteria strains *Burkholderia cenocepacia* AU1054 and *Ralstonia solanaceraum* GMI1000 available in the RefSeq database (Figure 5). The predicted genome size was 3.21 and 3.45 Mb for strains Lt-FCMA and Lt-F1 with 38.3x and 82.1x coverage and GC content of 52.3 and 51.7%, respectively. The average nucleotide identity among shared gene orthologs from the *Limnobacter* genomes was 92.5%. Analysis of genome annotations revealed genes for lithotrophic sulfur oxidation (Sox genes), while genes for carbon fixation to enable autotrophic growth were not evident. This observation was consistent with physiological characterization that indicated that *Limnobacter* strains did not grow in the absence of exogenously supplied organic carbon, and these strains may thus be mixotrophic. The virulence factors in the *Limnobacter* genomes include the common Proteobacterial factors as well as genes with homology to a beta-lactamase involved in antibiotic resistance and a salicylate synthetase. In addition, several genes were identified as homologs of type III secretion proteins, although annotation of the corresponding open reading frames in RAST indicated that at least some of these genes may be mis-annotated flagellar genes and further work is needed to confirm this result.

The genome from the single *Stappia stellulata* strain was 4.42 Mb with 25.3x coverage and 65.3% GC. Like the *Limnobacter* strains this *Stappia* isolate was obtained from anemones collected in the field. The *Stappia* genome annotations indicate a complete pathway for oxidation of reduced sulfur compounds (Sox genes), as well as genes for utilizing carbon-monoxide and aromatic compounds as electron donors for growth. In addition to the common Proteobacterial virulence factors identified above the *Stappia* strain included proteins with significant similarity to ureases and antibiotic resistance proteins.

### No observed evidence for horizontal gene transfer between genome-sequenced bacterial lineages or with the *N. vectensis* host

Comparison of predicted phage-like or mobile genetic elements and high identity DNA sequences by BLASTN and BLASTP revealed no evidence of shared genetic elements with high nucleotide identity suggesting no recent horizontal gene transfer among bacterial lineages. Bacterial genome ORFs with BLASTP annotations from the NCBI NR Database were imported into MEGAN for taxonomic binning using the Lowest Common Ancestor algorithm (Huson et al., 2007). As expected, the majority of the assigned ORFs binned within the assigned Class of the bacterial isolate. In addition, ORFs within the *Pseudomonas* and *Rhizobium* strains were classified as viral in origin consistent with identification of several likely bacteriophage and phage-related genes. Surprisingly, four ORFs from the *P. oleovorans* strains were classified as being of *N. vectensis* origin. While it may be possible that genes have been horizontally transferred between the anemone and bacteria, in either direction, the most parsimonious explanation is that the *N. vectensis* reference genome is contaminated with *Pseudomonas* DNA incorrectly annotated as cnidarian as recently described by Artamonova and Mushegian (2013). To examine this further, nucleotide BLAST of *P. oleovorans* and *N. vectensis* genomes revealed *Pseudomonas* DNA on 101 *N. vectensis* genome scaffolds that were, on average about 20 kb shorter than the average and contain almost 80% ambiguous nucleotides with an average GC of 60% similar to that of the sequenced *P. oleovorans* (64.1–64.9%). BLASTN of the *N. vectensis* genome with the *Rhizobium, Limnobacter*, or *Stappia* genomes sequenced in this study revealed an additional 6, 4, and 3 *N. vectensis* genome scaffolds that likely derive from bacterial contaminants, respectively. All *N. vectensis* scaffolds identified with likely bacterial sequence by this approach are indicated in Supplementary Table 1. On average proteins shared by the *P. oleovorans* strains reported in this study, and identified as originating from *Pseudomonas* in the *N. vectensis* genome shared 75% amino acid similarity (range 25–98%).

### Preparation of a *N. vectensis* laboratory holobiont metatranscriptome

Because analysis of 16S rRNA genes in cDNA from field-collected *N. vectensis* revealed expressed ribosomal sequences from several candidate symbionts including Campylobacterales and *Endozoicomonas* ribotypes we sought to optimize protocols for further metatranscriptomic analysis. To this end we conducted a pilot metatranscriptome study to sequence enriched mRNA from RNA extracted from laboratory-raised anemones. After processing sequence data to remove ribosomal contamination and QC filtering (removal of Illumina adaptors and low complexity sequences; Table 5) sequences from the different treatments were combined yielding a final total of 529,425 sequence pair units. We noted that of the treatments examined, the RNA sample processed with the MICROBEnrich/MICROBExpress+mRNA-only kits for rRNA depletion performed best in terms of sequence yield with 246,506 out of 653,926 sequences identified as putative mRNAs (37.7%) compared to the unprocessed control (8.24%). In absence of replication, these observed differences between approaches are purely anecdotal. Data from this initial screen, and from published studies (He et al., 2010; Stewart et al., 2010), supported adoption of the mRNA-only + Microbe Express/Enrich protocol for future work and has yielded similar proportions of sequences and successful enrichment of bacterial mRNAs among complex targets (Penn et al., 2014).

**Table 5:** **Processing of ***N. vectensis*** metatranscriptomes to remove ribosomal RNAs and low-quality sequences**.

**Process performed on sample for rRNA depletion^a^**	**Initial**	**Reads removed during QC after identification to categories of rRNA or low quality sequence**					**Final**
	**Total sequence pairs**	**Large subunit rRNA**	**Small subunit rRNA**	**5S or ITS rRNA**	**Tandem repeats**	**Illumina adaptors**	**Non-ribosomal sequence pair units^b^(% Initial)**
Poly(A)purist + RNaseH	1,433,848	987,049	418,609	1358	701	12,824	13,307 (0.92%)
Poly(A)purist + mRNAonly	969,506	429,858	208,186	753	24,171	195,907	110,631 (11.40%)
Poly(A)purist + RNaseH + mRNAonly	165,5964	1,124,016	307,443	1454	1659	153,249	68,143 (4.11%)
Poly(A) purist+ MICROBEnrich+ MICROBExpress + mRNAonly	653,926	368,740	33,371	475	3242	1592	246,506 (37.7%)
Poly(A) purist + mRNAonly +DSNuclease	107,964	95,649	12,188	1	2	7	117 (~0.001%)
Total RNA unprocessed	1,100,418	767,151	235,462	741	3793	2550	90,721 (8.24%)
Total analyzed	5,921,626	3,772,463	1,215,259	4782	33,568	366,129	529,425 (8.9%)

*ITS, Internal Transcribed Spacer*.

a *The processes implemented for depletion of rRNA and non-bacterial mRNA included treatment of total RNA with: (1) RNAseH after hybridization with DNA oligos targeting specific conserved regions of rRNA - RNAseH is an endonuclease that specifically degrades RNA in RNA:DNA hybrids, (2) the MICROBEnrich^TM^ Kit (Ambion Part No. AM1901) and MICROBExpress^TM^ Kit (Ambion Part No. AM1905), a pair of kits that rely on a novel capture oligo hybridization protocol to selectively remove eukaryotic rRNA and Bacterial rRNA respectively, (3) mRNAOnly reagent (Epicenter), an endonuclease-based method that selectively degrades RNAs with 5′-monophosphates, (4) duplex-specific nuclease (DSN) treatment after hybridization with DNA oligos targeting specific conserved regions of rRNA. DSN specifically degrades dsDNA and DNA in DNA:RNA hybrids, and (5) Poly(A)purist kit that relies on use of oligo(dT) cellulose to preferentially bind Poly(A) tails of eukaryotic mRNA. Treatments were used in combinations specified above and kits were implemented according to the manufacturer's protocols*.

b *A sequence pair unit can be one of three things: (1) A sequence pair whose ends have both made it through filtering. (2) A pair of sequences merged into one sequence because of shared overlapping sequence. (3) A pair of sequences clipped to one sequence because of adaptor contamination*.

### Assembly and annotation of holobiont metatranscriptome sequences

Assembly of sequence pair units yielded 7296 contigs where 3422 and 2809 were classified as Eukaryotic and *N. vectensis*, respectively, through comparison to the NCBI non-redundant protein database. Ten contigs assigned to the bacteria were analyzed more closely by BLASTN and BLASTX. One assembled contig of 528 nt derived from 12 sequence pairs had 97% nucleotide identity to the *Vibrio campbellii* outer membrane protein OmpU (average coverage of 2.46X). Remaining contigs were revealed to be *N. vectensis*-like or revealed no higher than 40% amino acid identity to predicted proteins, precluding annotation.

### Taxonomy of individual metatranscriptome sequences

**Figure 6:**
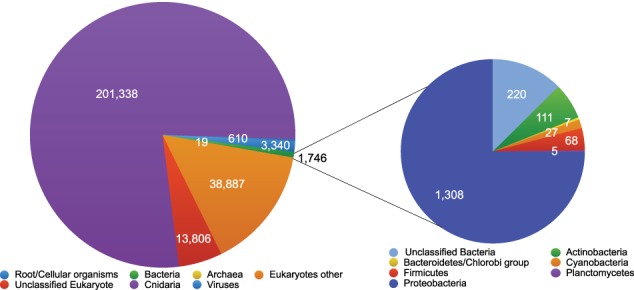
**Diversity of metatranscriptome sequences identified by BLASTX against the NCBI non-redundant protein database**. Identified sequences were imported into the MEGAN software package and binned taxonomically using the least common ancestor algorithm (Bit score >40.0). Matches to bacterial phyla are presented.

Individual sequence pair units were compared against the NCBI non-redundant protein database using BLASTX and slightly less than half of the sequences shared significant similarity with database proteins (i.e., 259,746 database matches) and were assigned to taxonomic groups using MEGAN. Consistent with assembled contigs, the majority of sequences with database matches were “Cnidarian” corresponding to the host anemone taxonomy (77.5% of assigned sequences) (Figure 6). The other top assignments were Metazoan taxa (12.6% of assigned sequences), Opisthokonta (2.2%), and Eukaryota (5.6%) suggesting that >90% of the expressed non-ribosomal sequences from the holobiont derived from the host anemone. Sequences similar to microbial eukaryotes each corresponded to < 0.15% of sequences. Of the 1746 sequences annotated as bacterial (0.67% of sequences with database matches), 1308 corresponded to Proteobacteria (75%), followed by unclassified bacteria (13%), Actinobacteria (6.4%) and Firmicutes (3.9%) (Figure 6).

### Recruitment of metatranscriptome sequences to bacterial isolates

Metatranscriptome sequences were mapped as unpaired reads to the sequenced and annotated genomes of the 10 cultured *N. vectensis* associated bacteria. Twenty-four gene families (COG/NOG) from the *Limnobacter* genomes matched sequences in the metatranscriptome with 95-100% sequence identity over at least 200 bp of consensus sequence (Table 6). Expressed ORFs from the *Pseudomonas, Rhizobium* or *Stappia* genomes were not detected by this approach. The most highly represented *Limnobacter* gene among metatranscriptome sequences (3.6X coverage of a 489 bp ORF with 100% identity between the consensus sequence and the Lt-FCMA genome) was predicted as derived from the phasin protein family (NOG45042), a group of proteins responsible for the synthesis and structure of Poly 3-hydroxyalkanoate (PHA) granules (Table 6). Expression of a predicted PHA synthase (COG3243) that participates in PHA granule formation was also detected in the metatranscriptome (99% sequence identity over a 202 bp region of a 1794 bp ORF). Predicted functions for other *Limnobacter* ORFs with metatranscriptome matches include a phosphatase (COG3211), a transporter for phosphate (COG0226), a transporter for iron (COG1629), a TonB-dependent siderophore receptor (COG4774), and a flagellar motility protein (COG2063) (Table 6).

**Table 6:** **Summary of open reading frames from the ***Limnobacter thiooxidans*** genomes that recruited >200 bp of sequence data from the pilot metatranscriptome**.

**Predicted function (COG/NOG)**	No. of reads mapped^*^	***Limnobacter* str. FCMA**	***Limnobacter* str. F1**
Phasin family protein (NOG45042)	22	fig|6666666.35448.peg.16	fig|6666666.35449.peg.2832
Predicted phosphatase (COG3211)	6	fig|6666666.35448.peg.473 fig|6666666.35448.peg.1889	fig|6666666.35449.peg.2393
Outer membrane receptor proteins, mostly Fe transport (COG1629)	6	fig|6666666.35448.peg.2773 fig|6666666.35448.peg.3175	
Hypothetical	6		fig|6666666.35449.peg.589
ABC-type phosphate transport system, periplasmic component (COG0226)	4	fig|6666666.35448.peg.960	
Outer membrane protein and related peptidoglycan-associated (lipo)proteins (COG2885)	4	fig|6666666.35448.peg.1998	fig|6666666.35449.peg.630
Hypothetical	3	fig|6666666.35448.peg.3668	
Outer membrane protein (porin) (COG3203)	3		fig|6666666.35449.peg.1575
Uncharacterized protein conserved in bacteria (COG2908)	3	fig|6666666.35448.peg.3259	
Galactose oxidase (NOG69967)	2	fig|6666666.35448.peg.1616	
NOG268346	2		fig|6666666.35449.peg.1518
Acetyl-CoA acetyltransferase (COG0183)	2	fig|6666666.35448.peg.3397	
Flagellar basal body L-ring protein (COG2063)	2	fig|6666666.35448.peg.1203	
Acyl-CoA dehydrogenases (COG1960)	2		fig|6666666.35449.peg.422
ABC-type amino acid transport/signal transduction systems, periplasmic component/domain (COG0834)	2		fig|6666666.35449.peg.3136
Outer membrane receptor for monomeric catechols (TonB dependent siderophore receptor) (COG4774)	2	fig|6666666.35448.peg.3444	
FOG: WD40-like repeat (COG1520)	2	fig|6666666.35448.peg.3042	
Guanylate kinase (COG0194)	2	fig|6666666.35448.peg.2698	
DNA uptake lipoprotein (COG4105)	2	fig|6666666.35448.peg.3392	
Ribosomal protein L13 (COG0102)	2	fig|6666666.35448.peg.475	
Poly(3-hydroxyalkanoate) synthetase (COG3243)	2	fig|6666666.35448.peg.3396	
Transcriptional regulator (COG1309)	2		fig|6666666.35449.peg.2988
Nucleoside-diphosphate-sugar epimerases (COG0451)	2	fig|6666666.35448.peg.3380	
Uncharacterized protein conserved in bacteria (COG3490)	2	fig|6666666.35448.peg.1509	
Hypotheticals	2 ea.	fig|6666666.35448.peg.1217 fig|6666666.35448.peg.2235	fig|6666666.35449.peg.2646 fig|6666666.35449.peg.3297 fig|6666666.35449.peg.3370 fig|6666666.35449.peg.3550

* *Paired sequences from the metatranscriptome were mapped as single reads to the open reading frames of the two sequenced Limnobacter associates. Mapping results are reported where the consensus sequence ≥200 bp. Open reading frames were annotated using the COG and NOG subsets of the eggNOG database (version 3.0)*.

## Discussion

Symbiotic bacteria associated with cnidarians have recently become focal points for research to understand the roles of these microorganisms in the health and disease of their hosts. As a model cnidarian, *N. vectensis* and its bacterial associates represent a tractable system for examining potential mechanisms for microbial persistence in the holobiont. While previous research had provided evidence of microbial contamination of the *N. vectensis* genome (Starcevic et al., 2008; Har, 2009; Artamonova and Mushegian, 2013) the research presented here is the first to document the diversity of microbial associates in wild and laboratory raised *N. vectensis* and to describe the physiological and genomic variation of culturable microbes associated with this anemone. Through analysis of 16S ribosomal RNA clone libraries we have observed that bacterial OTUs of a novel Campylobacterales spp. as well as *Endozoicimonas elysicola* are associated with *N. vectensis* in geographically distinct salt marshes, while a novel Spirochete OTU and *Pseudomonas oleovorans* have been observed in *N. vectensis* collected from both the field and the laboratory. Species-specific PCR indicates that these populations may persist in the holobiont of laboratory-acclimated *N. vectensis* for at least 9 months (Figure 4) after transfer from their natural salt marsh habitat. Similarly, isolation of *Limnobacter* strains from both field-collected and laboratory-raised anemones suggests these strains are also able to persist in the holobiont from the field to laboratory, although their absence from clone libraries suggest that these are not dominant taxa in either environment. Isolation of *Stappia* strains from *N. vectensis* collected from different Massachusetts marsh sites, and *R. radiobacter* strains from laboratory-acclimated *N. vectensis* over a 2 year timeframe suggests these two populations may be stable associates of *N. vectensis* in the salt marsh and laboratory environments, respectively. Thus, we hypothesize that these populations are *N. vectensis* symbionts due to their apparently stable association with the anemone (de Bary, 1879; Chaston and Goodrich-Blair, 2010).

### *N. vectensis*-associated bacteria are closely related to coral and sponge associates

Association of strains closely related to the *N. vectensis* symbionts described in this study with other marine Cnidarians or Porifera suggest that these bacteria may be adapted to life in association with early diverging Metazoan hosts (species in the phyla Cnidaria and Porifera). The Campylobacterales population that is the most-abundant bacterial associate of *N. vectensis* is most closely related to an uncultured sequence from the Caribbean coral *Montastraea (Orbicella) faveolata* (97% 16S rRNA identity) (Sunagawa et al., 2009). Similarly, the novel Spirochete OTU is most closely related (92.8% 16S rRNA identity) to a deep-sea coral clone (Kellogg et al., 2009). *Pseudomonas oleovorans (pseudoalcaligenes)* (99.6% 16S rRNA identity) has been isolated from the marine sponge *Ianthella bastain* (Cervino et al., 2006), is widely distributed in the terrestrial and marine environment (Nishino and Spain, 1993; Quinteira et al., 2005) and is regarded as an opportunistic pathogen of humans (Gilardi, 1972) and other animals (Yamamoto et al., 2000). Finally, recent studies have shown that *Endozoicomonas elysicola*-like bacteria are associated with marine invertebrates including a wide diversity of Cnidarians. Sequences with high ribotype identity (≥97%) with *Endozoicomonas elysicola* have been found in three sea anemones: *N. vectensis* (this study), *Metridium senile* (Schuett et al., 2007) and *Anthopleura midori* (Du et al., 2010), in addition populations of *Endozoicomonas* spp. are found at high proportion across multiple types of corals (Raina et al., 2009; Sunagawa et al., 2010; Yang et al., 2010; Morrow et al., 2012; Pike et al., 2013; Bayer et al., 2013a,b; Morrow et al., 2014; Neave et al., 2014) and other marine invertebrates (e.g., the sea slug *Elysia ornate*, Kurahashi and Yokota, 2007).

The culturable symbionts analyzed by genome sequencing also share close relation to sequences and isolates recovered from coral and sponge holobionts. *Stappia stellulata* strains have been recovered from a wide diversity of marine invertebrates (Boettcher et al., 2000; Weber and King, 2007) and the isolate derived from *N. vectensis* in this study matched a ribotype found in a Black Band Diseased coral *Siderastrea siderea* (DQ446087) (Sekar et al., 2006). The sequenced strains of *R. radiobacter* are closely related to the agent of crown-gall disease in plants, which was formerly identified as and still commonly called *Agrobacterium tumefaciens* (Young et al., 2001). A closely related sequence to *R. radiobacter* isolates from our study was recovered from a survey of coral reef bacterioplankton (>98% to HQ443405) (Nelson et al., 2011) and appears to be widespread in marine environments (Engelhardt et al., 2013). Other members of the genus *Rhizobium* are well known for symbiotic nitrogen fixation in plants and are of emerging interest due to their potential role in nitrogen fixation within the coral holobiont (Lema et al., 2012). Bacterial isolates from the genus *Limnobacter* have been found in diverse environments including freshwater lake sediments, the surface waters of the Baltic and Mediterranean Seas, a volcanic deposition in Japan, soils at a coal-mining site (Spring et al., 2001; Lu et al., 2011; Vedler et al., 2013; Poncelet et al., 2014). While no published studies indicate animal association is common in this taxonomic group we note that a strain of *Limnobacter thiooxidans* sharing >98% rRNA identity with the strains described in this study was isolated from the sponge *Haliclona simulans* in the South China Sea (FJ999570, unpublished study) and symbioses have been documented within other genera within the family Burkholderiaceae e.g., Kim et al. (2013).

### Potential microbial activities in the *N. vectensis* holobiont

Guided by the diversity of described marine microbial symbioses we can pose several tentative hypotheses based on our data regarding the activities of microorganisms that associate with the *N. vectensis* holobiont. First, we suggest that the associations between the bacterial isolates characterized in this study and the anemone are facultative based on their ease of cultivation, and the diversity and size of genomic repertoires suggesting that these particular strains have not experienced overall genome size reduction that is characteristic of more obligate symbioses. In contrast, symbionts observed via 16S rRNA clone libraries that have resisted cultivation in this study may represent more fastidious or obligate associations and remain attractive targets for further work to uncover the mechanisms of association and persistence. Based on our current phylogenetic, genomic, and pilot-scale metatranscriptomic data we suggest several activities that may mediate survival and persistence of bacterial populations in the *N. vectensis* holobiont, namely (1) the use of alternative forms of energy generation (mixotrophy), (2) scavenging of nutrients (P and Fe), (3) storage of carbon, and (4) expression of mechanisms to resist chemical stressors. All of these factors have been identified as relevant to other host x microbe associations that are discussed in more detail below.

#### Sulfur oxidation as a potential form of mixotrophy in *N. vectensis* microbiota

Several members of the *N. vectensis* holobiont described in this study either contain genes for sulfur oxidation, or are in a phylogenetic lineage that contains species that are known sulfur compound oxidizers. The closest culture-characterized relatives of the Campylobacterales OTU, numerically dominant in anemones collected from the salt marsh habitat, includes a sulfur-oxidizing chemolithoautotroph (*Sulfurovum lithotrophicum*). Chemoautotrophic εpsilon-Proteobacteria that use reduced sulfur compounds as electron donors, are found in symbiotic associations with animals in environments exposed to high fluxes of reduced sulfur compounds such as hydrothermal vents and salt marshes (Madrid et al., 2001). Genomes from *Limnobacter thiooxidans* and *Stappia stellulata*, isolated from field-collected anemones, reveal ORFs annotated as genes for sulfur oxidation (sox) but not autotrophic carbon fixation, suggesting these species may be able to utilize reduced sulfur compounds to supplement heterotrophic growth in the anemone holobiont. Despite observation of sox genes in the genome of *S. stellulata*, to our knowledge mixotrophic growth has not been reported for other strains of the species (Buchan et al., 2001; Weber and King, 2007). *Limnobacter thiooxidans* was originally described as a mixotroph (Spring et al., 2001) and this trait is observed in other members of the genus (Lu et al., 2011). Fluxes of sulfide are characteristic of the anemone's salt marsh habitat (Howes et al., 1985) and utilization of this alternative source of electrons for energy-generation may promote persistence in the host during times of nutrient scarcity. In addition, oxidation of reduced sulfur compounds in the *N. vectensis* holobiont could increase holobiont fitness through detoxification of internal sulfide, or by fueling autotrophic-production of microbial biomass as an internal food supply, as has been demonstrated in other marine microbe symbioses (Childress et al., 1991; Krueger et al., 1996; Freytag et al., 2001; Dubilier et al., 2008). Further work is warranted to investigate whether mixotrophic sulfide oxidation may play a similar role in the *N. vectensis* holobiont in its native salt marsh range.

#### Scavenging nutrients

The importance of the nutrients iron and phosphorous within the microbiota is suggested by analysis of genomes and metatranscriptomes. All *N. vectensis* associated bacterial genomes revealed genes for the biosynthesis of high affinity iron-binding compounds (siderophores); such compounds are well-established as host association factors due to competition between the host and microbiota for bioavailable iron. Metatranscriptome sequences mapped with high stringency to *Limnobacter* ORFs predicted to encode proteins for nutrient scavenging including two types of siderophore receptors (COGs 1629 and 4774) as well as an alkaline phosphatase and a phosphate transporter (COGs 3211 and 0226) that enable cleavage of phosphate groups from organic compounds followed by uptake. Iron and phosphorous are both essential nutrients and enrichment/expression of nutrient transporters has been shown to correlate to environmental stress for the respective nutrient (Coleman and Chisholm, 2010; Harke and Gobler, 2013). As siderophores promote the survival of pathogens during infection they are widely identified as virulence factors; yet these compounds have been shown play much broader ecological roles by controlling the dynamics of plankton populations in low-iron ocean regions and mediating ecological interactions among coastal bacterioplankton (Cordero et al., 2012) and coral reefs (Kelly et al., 2012).

#### Resource storage

The *Limnobacter* ORF with the highest coverage in the *N. vectensis* holobiont metatranscriptome corresponded to a phasin protein in the gene family NOG45042, which regulates biosynthesis of Poly 3-hydroxyalkanoate (PHA) granules for intracellular storage of carbon (Table 6). A second ORF detected in the metatranscriptome corresponded to a PHA synthetase (COG 3243). PHA granules have recently been determined to play a critical role in symbiosis of a Betaproteobacterial species (genus *Burkholderia*) with the bean bug *Riptortus pedestris* (Kim et al., 2013). The phasin protein was more highly expressed in bean bug-associated bacteria than in cultures of the *Burkholderia* strain (Kim et al., 2013). Evidence of the role of PHA in symbiosis was provided and when genes for PHA synthesis were inactivated by mutagenesis resulting in a reduced density of the *Burkholderia* population within the bean bugs which, in turn, became more vulnerable to osmotic, oxidative, nutrient, and temperature perturbations (Kim et al., 2013). This work suggests that PHAs mediate the persistence of bacterial cells under various environmental stresses and it is possible that PHA granules may play a similarly important role in *Limnobacter's* acclimation and persistence within the anemone holobiont.

#### Resistance to chemical stressors

A *Vibrio* OmpU-like protein was the sole bacterial transcript assembled from the *N. vectensis* holobiont metatranscriptome. OmpU, an outermembrane porin, has been shown to modulate host and symbiont interaction in several vibrios, mediating colonization of the mutualist *V. fisheri* (Aekersberg et al., 2001) and virulence of the pathogens *V. splendidus* (Duperthuy et al., 2010) and *V. cholerae* (Provenzano and Klose, 2000). Loss of OmpU function in *V. splendidus* was associated with higher sensitivity to host-derived antimicrobial peptides (Duperthuy et al., 2010). Porins were also among the predicted cell-wall and membrane *Limnobacter* ORFs detected among metatranscriptome sequences (Table 6). Antibiotic resistance in bacteria is mediated by selective permeability at the cell wall and membrane, which is mediated by porins as well as efflux pumps that control penetration of toxicant compounds (e.g., antibiotics, antimicrobial peptides) to the interior of the bacterial cell (Yeaman and Yount, 2003; Piddock, 2006). Cnidarians are known to make a diverse array of antimicrobial compounds, and it has been recently shown that the model cnidarian *Hydra* regulates the composition of its microbiota through antimicrobial activity (Franzenburg et al., 2013a,b). Selective permeability of the bacterial cell wall may point to the importance of bacterial acclimation to the chemical environment of the anemone for persistence.

## Conclusion

We have used an integrated approach of cultivation independent microbiota surveys, strain isolation, genome sequencing, physiological characterization, and holobiont metatranscriptomics to explore the diversity and activity of the microbiota associated with *N. vectensis* in both the field and laboratory setting. This work has enabled preliminary insights into both the biodiversity of the *N. vectensis* holobiont over space and time and the mechanisms by which bacteria may persist in association with the *N. vectensis* host. Predicted activities of *Limnobacter* ORFs detected in the *N. vectensis* metatranscriptome parallel activities noted as important in other established symbioses, including nutrient scavenging, selective permeability of the cell wall/membrane and PHA granule formation which may play a role in bacterial resistance to holobiont-associated stresses. In addition, mixotrophic use of reduced sulfur compounds as electron donors is a potential activity of bacteria that appeared to be stably associated with *N. vectensis* across multiple filed sites (Campylobacterales OTU) and genes for this were detected in *Limnobacter* and *Stappia* isolates recovered from natural populations of *N. vectensis* in sulfide-rich salt marsh habitats. To better understand bacterial acclimation and persistence within cnidarian holobionts, further work should focus on organisms recovered from their natural habitats with the additional goal to elucidate activities of microbial populations that resist culturing and may reflect more obligate associations within the holobiont.

### Nucleotide accession numbers

Nucleotide Accession Information: The sequences obtained in this study have been deposited to Genbank under accession numbers HQ189546 to HQ189745. Genome sequences are deposited under BioProject Number PRJNA281237 and annotations are referenced by strain name and are publically available via the RAST server.

### Conflict of interest statement

The authors declare that the research was conducted in the absence of any commercial or financial relationships that could be construed as a potential conflict of interest.
