# Supplementary material for: Microbial diversity and activity in the Nematostella vectensis holobiont: insights from 16S rRNA gene sequencing, isolate genomes, and a pilot-scale survey of gene expression
Source: Front Microbiol. 2015 Sep 2;6:818. doi: 10.3389/fmicb.2015.00818 (PMC4557100; doi:10.3389/fmicb.2015.00818)
Supplement: Supplementary file 1 [file Table1.DOCX]

**Supplementary Table 1 –** List of scaffolds from the JGI assembly of *Nematostella vectensis* that likely are bacterial contaminants based on blastN alignments to the 10 sequenced *N. vectensis*  associated isolates

jgi|Nemve1|156831|e_gw.12797.7.1

jgi|Nemve1|156351|e_gw.10270.1.1

jgi|Nemve1|144785|e_gw.1158.3.1

jgi|Nemve1|3316|gw.7009.1.1

jgi|Nemve1|144756|e_gw.1152.1.1

jgi|Nemve1|148716|e_gw.2574.7.1

jgi|Nemve1|60966|gw.2574.4.1

jgi|Nemve1|148718|e_gw.2574.12.1

jgi|Nemve1|62952|gw.1345.3.1

jgi|Nemve1|68884|gw.9505.6.1

jgi|Nemve1|68384|gw.9505.4.1

jgi|Nemve1|156025|e_gw.9580.4.1

jgi|Nemve1|156293|e_gw.10013.4.1

jgi|Nemve1|9216|gw.10013.1.1

jgi|Nemve1|157196|e_gw.14966.6.1

jgi|Nemve1|145135|e_gw.1325.6.1

jgi|Nemve1|152225|e_gw.4762.1.1

jgi|Nemve1|61676|gw.3793.4.1

jgi|Nemve1|149172|e_gw.2834.1.1

jgi|Nemve1|145019|e_gw.1269.1.1

jgi|Nemve1|46382|gw.9892.1.1

jgi|Nemve1|144255|e_gw.981.2.1

jgi|Nemve1|155448|e_gw.8594.3.1

jgi|Nemve1|68429|gw.12503.2.1

jgi|Nemve1|153358|e_gw.6033.2.1

jgi|Nemve1|144964|e_gw.1244.3.1

jgi|Nemve1|68529|gw.1228.1.1

jgi|Nemve1|157171|e_gw.14678.2.1

jgi|Nemve1|157272|e_gw.15471.3.1

jgi|Nemve1|73238|gw.4271.1.1

jgi|Nemve1|78763|gw.18200.1.1

jgi|Nemve1|62331|gw.863.2.1

jgi|Nemve1|144785|e_gw.1158.3.1

jgi|Nemve1|3316|gw.7009.1.1

jgi|Nemve1|144756|e_gw.1152.1.1

jgi|Nemve1|148716|e_gw.2574.7.1

jgi|Nemve1|60966|gw.2574.4.1

jgi|Nemve1|148720|e_gw.2574.3.1

jgi|Nemve1|148718|e_gw.2574.12.1

jgi|Nemve1|68384|gw.9505.4.1

jgi|Nemve1|156025|e_gw.9580.4.1

jgi|Nemve1|9216|gw.10013.1.1

jgi|Nemve1|157196|e_gw.14966.6.1

jgi|Nemve1|157428|e_gw.16429.2.1

jgi|Nemve1|152225|e_gw.4762.1.1

jgi|Nemve1|61676|gw.3793.4.1

jgi|Nemve1|149172|e_gw.2834.1.1

jgi|Nemve1|46382|gw.9892.1.1

jgi|Nemve1|144255|e_gw.981.2.1

jgi|Nemve1|68429|gw.12503.2.1

jgi|Nemve1|156988|e_gw.13707.1.1

jgi|Nemve1|153358|e_gw.6033.2.1

jgi|Nemve1|144964|e_gw.1244.3.1

jgi|Nemve1|68529|gw.1228.1.1

jgi|Nemve1|157171|e_gw.14678.2.1

jgi|Nemve1|157272|e_gw.15471.3.1

jgi|Nemve1|73238|gw.4271.1.1

jgi|Nemve1|78763|gw.18200.1.1

jgi|Nemve1|62331|gw.863.2.1

jgi|Nemve1|144785|e_gw.1158.3.1

jgi|Nemve1|3316|gw.7009.1.1

jgi|Nemve1|144756|e_gw.1152.1.1

jgi|Nemve1|148716|e_gw.2574.7.1

jgi|Nemve1|60966|gw.2574.4.1

jgi|Nemve1|148720|e_gw.2574.3.1

jgi|Nemve1|148718|e_gw.2574.12.1

jgi|Nemve1|68384|gw.9505.4.1

jgi|Nemve1|156293|e_gw.10013.4.1

jgi|Nemve1|9216|gw.10013.1.1

jgi|Nemve1|145135|e_gw.1325.6.1

jgi|Nemve1|157428|e_gw.16429.2.1

jgi|Nemve1|152225|e_gw.4762.1.1

jgi|Nemve1|61676|gw.3793.4.1

jgi|Nemve1|149172|e_gw.2834.1.1

jgi|Nemve1|46382|gw.9892.1.1

jgi|Nemve1|144255|e_gw.981.2.1

jgi|Nemve1|155448|e_gw.8594.3.1

jgi|Nemve1|68429|gw.12503.2.1

jgi|Nemve1|156988|e_gw.13707.1.1

jgi|Nemve1|153358|e_gw.6033.2.1

jgi|Nemve1|68529|gw.1228.1.1

jgi|Nemve1|157171|e_gw.14678.2.1

jgi|Nemve1|73238|gw.4271.1.1

jgi|Nemve1|78763|gw.18200.1.1
